# Natural Bioactive Compounds Acting against Oxidative Stress in Chronic, Degenerative, and Infectious Diseases

**DOI:** 10.1155/2018/3894381

**Published:** 2018-12-03

**Authors:** Roberto Mattioli, Luciana Mosca, Angel Sánchez-Lamar, Italo Tempera, Ralf Hausmann

**Affiliations:** ^1^Department of Biology and Biotechnology, Sapienza University of Rome, 00185 Rome, Italy; ^2^Department of Biochemical Sciences “A. Rossi Fanelli”, Sapienza University of Rome, 00185 Rome, Italy; ^3^Department of Vegetal Biology, Facultad de Biología, Universidad de La Habana, Habana, Cuba; ^4^Fels Cancer Institute and Department of Microbiology, Temple University School of Medicine, USA; ^5^Molecular Pharmacology, RWTH Aachen University, Germany

Despite the great efforts made in primary prevention, many chronic and degenerative diseases such as cancer, neurodegenerative disorders, and cardiovascular and metabolic diseases are yet the most common causes of death and disability in industrialized countries. Many epidemiological studies suggest that a large consumption of fruit and vegetables can counteract the development of several chronic and degenerative diseases because of the presence of bioactive molecules in these foods. Indeed, natural bioactive compounds present in fruit and vegetables, such as polyphenols, flavonoids, anthocyanins, micronutrients, and vitamins, are well known for their antioxidant and anti-inflammatory roles and have been proposed as possible therapeutic agents against these diseases. Therefore, many efforts are focusing on the research of new bioactive molecules contained in plants and on the effects that these molecules have on human health. However, an alternative approach is the use of complex mixtures of bioactive molecules that appear to be very effective both *in vitro* and *in vivo*, possibly because of the synergistic action among different compounds, for instance, between polyphenols and other food components such as vitamins and minerals.

This special issue includes a collection of twelve contributions (reviews and original articles) regarding the effects that foods, plant extracts, and/or single isolated bioactive molecules have on some human pathologies. In particular, two reviews by I. Rady et al. and I. Suntar et al. keep attention on the antitumor effects of *Annona muricata* plant and on antitumor, antioxidant, antidiabetic, and antiobesity effects of *Citrus aurantium*, respectively. The reviews summarize current knowledge on the effects of both plants on some human diseases and discuss about their possible application as supplements, in various dietary formulations, in improved and affordable therapies.

The remaining contributions consist of original papers that focus their attention on isolated bioactive molecules or plant extracts tested for their antioxidant and anti-inflammatory capacities on several models of human diseases. In their paper, Y.-R. Li et al. described the isolation of bioactive molecules from *Litsea garrettii* plant as potential preventive agents against oxidative stress and inflammatory response in several cellular models. In particular, the authors identified twenty-one molecules, which were evaluated for their inhibitory effect on oxidative stress and inflammation, and identified 3-methoxy-5-pentyl-phenol (MPP) as an effective activator of Nrf2 and inhibitor of NF-*κ*B.

Another research by Y. Xue et al. regarded the evodiamine molecule, a natural alkaloid found in *Evodia rutaecarpa* fruits, able to attenuate P2X7-mediated inflammatory injury of human umbilical vein endothelial cells exposed to high free fatty acids. In particular, the evodiamine is able to ameliorate fatty acid-induced cytotoxicity and ROS production and to suppress the overexpression of P2X7 receptors, ligand-gated cation channels involved in inflammatory and immune responses.

The catalpol molecule, present in large amounts in the roots of *Rehmannia glutinosa*, a plant largely used in traditional Chinese medicine, is able to inhibit ROS production, DNA damage, and telomere shortening, activating the PGC-1a/TERT pathway and ameliorating atherosclerosis symptoms both *in vitro* and *in vivo*, as reported by Y. Zhang et al.

Finally, T. Zheng et al. reported the effects of salidroside, a bioactive molecule extracted from *Rhodiola rosea* plant, on high-fat diet-induced nonalcoholic fatty liver disease and investigated the underlying mechanism. Mice were fed with a high-fat diet or a normal diet and treated with salidroside or vehicle for eight weeks. Salidroside administration suppressed oxidative stress and inflammation in the liver and reduced the high-fat diet-induced obesity, blood glucose, and lipid deposition in the liver.

Not all molecules produced by plants exert beneficial effects. Indeed, some molecules or their excessive intake can have a negative impact on health. In this special issue, for example, Q.-S. Wang et al. reported that palmitate, the most common free fatty acid found in animals and plants, upregulates the activity of the epithelial sodium channel in the distal nephron and thus increases fluid volume and blood pressure.

The remaining original papers examined the protective effects against human diseases of functional foods or of complex mixtures of bioactive compounds extracted from plants.

A. Di Sotto et al., for example, investigated the antiviral activity of extracts from female inflorescences of *Humulus lupulus*, underlining the ability of the extract to interfere with different phases of viral replication and intracellular redox balance of infected cells. Moreover, the authors performed an extract characterization, identifying several phenolic compounds which could contribute to antiviral effect.

In another original research, F. Boasquívis et al. investigated the effects of *Paullinia cupana* extract, known as guarana, on *Caenorhabditis elegans* models for Alzheimer's and Huntington's diseases. In particular, the authors showed a delay of paralysis induced by beta-amyloid (A*β*_1-42_) in worms treated with guarana extract, identifying SKN1 and DAF-16 as two transcription factors involved in the protection mechanism. Moreover, the authors showed that the protective effect is associated with reduced intracellular ROS levels and proteasome activity modulation.

In the scientific literature, many papers show protective effects of broccoli, cauliflowers, Brussels sprouts, cabbages (all belonging to the family of Brassicaceae), and several types of seaweed on neurodegenerative diseases, cardiovascular pathologies, and metabolic disorders such as diabetes.

In this special issue, two original papers support these evidences. S. Dal et al. reported the beneficial *in vivo* effects of red cabbages on rats affected by type 2 diabetes. The authors showed a decrease of vascular oxidative stress and an increase of the vascular endothelial NO synthase expression. In the liver, the consumption of red cabbages decreased oxidative stress and activation of NF-*κ*B while increasing catalase activity and accumulation of Nrf2. In conclusion, red cabbage consumption improved the metabolic profile and hepatic function in rats.

The effects of seaweed *Gracilaria birdiae* were tested *in vivo* by J. A. C. Barros-Gomes et al. Mice fed with *Gracilaria birdiae* seaweed reduced weight gain and blood glucose levels compared to those in the control group. Moreover, the animals showed increased antioxidant capacity and enhanced glutathione reductase and catalase levels.

Taken together, the papers of this special issue corroborate scientific data, which show the importance of natural bioactive compounds on health and indicate that the plant kingdom is yet an important source of bioactive molecules, which may be used as prophylactic or therapeutic agents.

